# Application of novel and existing methods to identify genes with evidence of epigenetic association: results from GAW20

**DOI:** 10.1186/s12863-018-0647-2

**Published:** 2018-09-17

**Authors:** Angga M. Fuady, Samantha Lent, Chloé Sarnowski, Nathan L. Tintle

**Affiliations:** 1 Medical Statistics, Department of Biomedical Data Sciences, Leiden University Medical Center, Einthovenweg 20, 2333 Leiden, ZC Netherlands; 2 Department of Biostatistics, Boston University School of Public Health, 801 Massachusetts Avenue, Boston, MA 02118 USA; 3 Department of Mathematics and Statistics, Dordt College, Sioux Center, IA 51250 USA

**Keywords:** GAW20, DNA methylation, Single nucleotide polymorphism, Multimarker tests, Ascertainment, Epigenetics, Family data

## Abstract

**Background:**

The rise in popularity and accessibility of DNA methylation data to evaluate epigenetic associations with disease has led to numerous methodological questions. As part of GAW20, our working group of 8 research groups focused on gene searching methods.

**Results:**

Although the methods were varied, we identified 3 main themes within our group. First, many groups tackled the question of how best to use pedigree information in downstream analyses, finding that (a) the use of kinship matrices is common practice, (b) ascertainment corrections may be necessary, and (c) pedigree information may be useful for identifying parent-of-origin effects. Second, many groups also considered multimarker versus single-marker tests. Multimarker tests had modestly improved power versus single-marker methods on simulated data, and on real data identified additional associations that were not identified with single-marker methods, including identification of a gene with a strong biological interpretation. Finally, some of the groups explored methods to combine single-nucleotide polymorphism (SNP) and DNA methylation into a single association analysis.

**Conclusions:**

A causal inference method showed promise at discovering new mechanisms of SNP activity; gene-based methods of summarizing SNP and DNA methylation data also showed promise. Even though numerous questions still remain in the analysis of DNA methylation data, our discussions at GAW20 suggest some emerging best practices.

## Background

Over the last decade, the proliferation of genetic measurement technologies and computational capacity has spurned a host of methodological innovations in downstream data analyses. The popular and widely used genome-wide association study (GWAS) has had a large impact on our understanding of the heritable component of numerous phenotypes [1]. However, recent technological advances have brought us high-coverage genome sequence, genome-wide transcriptomics, copy-number polymorphism, proteomics, and metabolomics data. Genome-wide methylation data have also become available via “off-the-shelf” array-based measurement technologies. The increased accessibility of genome-wide methylation measurements, combined with an increased appreciation for the role of epigenetic variation in phenotypic expression, has ushered in a new era of methodological innovation.

Standard methods of methylation analysis use a general linear modeling approach to estimate the association between methylation levels and phenotypic variation. Typically, a separate model is fit for each cytosine-phosphate-guanine (CpG) site of interest. Despite the seemingly straightforward nature of this approach, it raises numerous methodological questions. For example, what are appropriate methods for accounting for relatedness and sample ascertainment? Are standard methods from GWAS (eg, kinship adjustment) sufficient? Or, are more sophisticated methods necessary? Although next-generation sequencing data and rare-variant analyses have brought multimarker analysis methods into the mainstream, we know little about the potential efficacy of these methods in the context of DNA methylation data. Yet another question of interest involves appropriate ways to integrate multiple data sources; for example, if methylation data (eg, DNA methylation probes) and genomic data (eg, single-nucleotide polymorphism [SNP] markers) are available, how best could this data be integrated to maximize the ability to explain phenotypic variation and identify candidate regions of the genome for future study?

As part of GAW20, 8 research groups were assigned to the Epigenetics: Gene Searching working group. These 8 groups from around the world met in San Diego, CA, USA to share and discuss their individual methodological innovations and analytic evaluations around the theme of identifying genes with evidence of epigenetic effects on phenotypes. In the following sections, we describe the 8 individual contributions, their methods and their results, and summarize 3 primary themes from our discussions.

## Methods

The Epigenetics: Gene Searching group at GAW20 consisted of 8 separate contributions. Groups analyzed a mix of real (6 contributions) and simulated (2 contributions) GAW20 data [2]. The real GAW20 data set consisted of both methylation data and high-density lipoprotein cholesterol (HDL-C)/triglyceride (TG) levels before and after treatment with fenofibrate, a drug used to assist in lowering cholesterol and TG levels [2]. A full set of SNP genotypes was also available for most individuals in the study. Real data was obtained from the Genetics of Lipid Lowering Drugs and Diet Network (GOLDN) study [3]. Data was simulated based on the real data and included 200 independently simulated sets of posttreatment TG and methylation levels, with hypothesized effects of genetic variation and methylation on TG/HDL-C levels [2]. The majority of groups used multimarker methods (6 of 8 contributions) and/or candidate gene/marker approaches (5 of 8 contributions). We now briefly describe the methods used by each of the 8 separate contributions. In most cases, additional information about each contribution, beyond that presented here, can be gleaned by reading the related publications for each individual contribution; citations, where available, are provided.

Fuady et al. [4] aimed to investigate the relationship between change in TG and change in methylation after treatment. They focused on strategies of ascertainment correction for families. From the real data set, they analyzed 421 individuals who had data on both TG and methylation levels. They defined the change in methylation at each CpG site as the difference between methylation values at baseline and follow-up. Change in TG was computed similarly, as the difference between the mean log-transformed TG at baseline and posttreatment. They evaluated 2 ascertainment bias correction methods and compared them to a naïve approach that ignores ascertainment.

Huisman et al. [5] evaluated the performance of novel gene-based test association methods on GAW20 simulated genome-wide data (*N* = 670) as compared to single-marker tests. They used the *lmekin* function from the *coxme* package in R [6] to predict the change in log-transformed TG levels from baseline to follow-up, adjusting for familial relationships. Then they regressed the residuals from the previous model on genetic variants and methylation scores. They defined sets of candidate genes and, for each gene, created different scores by aggregating beta coefficients or *p* values for SNPs or DNA methylation probes. They assessed statistical significance of genes using permutations.

Lent et al. [7] proposed a new method, GlobalP, to detect differentially methylated regions (DMRs), regions enriched for association with a phenotype, and compared this new method to 3 existing methods: Bumphunter [8], comb-p [9], and DMRcate [10]. Lent et al. applied Bumphunter to a subset of unrelated individuals (pretreatment *N* = 176, posttreatment N = 176) and all other methods to all individuals with both genotype and methylation data (pretreatment *N* = 679, posttreatment *N* = 403). Two epigenome-wide association studies (EWAS), with pretreatment and posttreatment TG as the outcomes, were performed in the related sample, adjusting for empirical kinship estimated from genotypes, to calculate summary statistics for input into DMRcate, comb-p, and GlobalP.

Sarnowski et al. [11] performed parent-of-origin effect association analyses of genetic variants with TG (pretreatment TG, posttreatment TG and pretreatment and posttreatment TG difference) followed by association analyses with DNA methylation probes in the real GAW20 family data set. From the real data, they analyzed 823 genotyped individuals from 173 families (715,787 SNPs). They used a linear regression approach implemented in the Quantitative Transmission Disequilibrium Test (QTDT) software [12] to detect parent-of-origin associations of genetic variants with TG. Then, they investigated if the detected parent-of-origin effects were associated with TG-associated CpG sites using linear mixed effect models. Finally, they performed causal inference tests [13] to assess whether methylation mediated the observed parent-of-origin effects.

Wang et al. [14] explored 2 methods to investigate the gene-based changes of DNA methylation and the associations with lipid changes induced by treatment: the median methylation level test (MMLT [15]) and the sequence kernel association test (SKAT [16]). They analyzed 446 individuals at 423,180 CpG sites. The median methylation level of all the CpG sites within a region was calculated to summarize the typical methylation level of the gene. They performed linear mixed effect regression models to investigate the association between change in methylation and change of either TG or HDL-C after 3 weeks of intervention. Then they used the SKAT to evaluate the association of change in TG or HDL-C with a set of CpG sites.

Wu et al. [17] extended the adaptive sum of powered score (aSPU) test for methylation data [18]. The sum of powered score (SPU) test is a score based test with 2 parameters: a power index and component weights. Special cases include the Burden test [19] when the power index is 1 and the SKAT test [16] when the power index is 2. The aSPU test is an extension to SPU that does not require choosing a power index, but still requires the choice of component weights. Wu et al. proposed using the inverse variance of CpG sites as the weights for aSPU. Wu et al. modeled the association between pretreatment methylation and TG using gene-based aSPU tests and compared this to a standard EWAS implemented in GMMAT (generalized linear mixed model association test) [20]. All models used the related sample, adjusting for empirical kinship estimated from genotypes.

Xu et al. [21] proposed new methods to identify SNP markers and CpG variants associated with a phenotype of interest (TG or HDL-C) using an iterative regression or an extreme value approach. The iterative regression strategy is a single-marker (single-SNP or single-CpG site) method, whereas the extreme value strategy is a gene-based method. They used 418 cases and 687 control individuals and examined 467,225 SNPs. Combinations of the selected SNP/CpG sites (iterative regression strategy) or candidate genes (extreme values strategy) were tested using a score test proposed by Chapman et al. [22]. To potentially improve power, a hybrid approach of these strategies was also considered. They compared the performance of each approach with the correlated method [23].

Zhao et al. [24] focused their investigation on gene-based association between DNA methylation and lipid-level changes in chromosomes 11 and 19. They explored 2 complementary region-based association tests. They used 420 related individuals for whom pretreatment methylation data were available and, in separate analyses, 176 unrelated individuals. Methylation data on chromosomes 11 and 19 were grouped into 1484 and 1698 genes, respectively, and transformed using the logit function. The principal components of explained variance (PCEV [25]) and a variance component (VC) score test [26] were used to analyze the unrelated individuals. Family data were analyzed via an extension of a VC-score test [26].

## Results

### Theme 1: Family versus unrelated individuals

Six contributions in our group specifically explored the family data to perform association analyses (Fuady et al..., Lent et al., Sarnowski et al., Wang et al., Wu et al., Zhao et al). In the contribution of Fuady et al., family data was used to investigate the association between lipid changes and methylation changes after treatment using various family-based ascertainment correction approaches. Zhao et al. employed family structure to identify gene-based association between chromosomes 11 and 19 DNA methylation sites and change in lipid levels. Parent-of-origin effect association analyses were performed by Sarnowski et al., using the family data. Wang et al. explored 2 independent methods to investigate the association between changes in methylation and drug response in families. Wu et al. used family data to assess the relationship between baseline methylation and TG levels. Lent et al. compared various methods to analyze the association between DMRs and TG level using family data. In all contributions, empirical kinship was used to adjust for family structure.

### Ascertainment correction

Ascertainment correction plays a role not only for families but also for unrelated individuals who were selected from the pedigree. The available data set in GAW20 is based on the GOLDN study, which recruited families with at least 2 coronary heart disease events and a family risk score of 0.5 or higher [2, 27]. The multiple-case family design is enriched for the outcome variable (primary phenotype). Some work has been done when modeling the primary phenotype to address the ascertainment bias [28]. When modeling the secondary phenotype, it is also necessary to correct for the ascertainment, especially when the primary and the secondary phenotype are correlated [29].

Fuady et al. explored 2 existing statistical methods to take ascertainment into account for multiple-case families. The first method is the secondary phenotype approach [30]. In this approach, the ascertainment process is corrected using the retrospective likelihood and joint model between the primary and secondary phenotype. They choose metabolic syndrome (MetS) as the primary phenotype and methylation as the secondary phenotype. The second approach is an ascertainment correction based on proband as implemented in SOLAR (Sequential Oligogenic Linkage Analysis Routines) [31]. The correction was performed by conditioning the likelihood of each pedigree with the trait value of the proband [32].

In the contribution of Zhao et al., the ascertainment correction was implemented by constructing principal components (PCs) of genome-wide methylation levels using 2000 probes randomly sampled from all chromosome. PCs were used to adjust for known and unknown confounders. Moreover, it is also useful to improve the validity of the VC-score test. The top 4 methylation-derived PCs were used as an additional covariate in the model. An extended VC-score test [26] was used to incorporate the family structure in the data set. This approach decomposes the total variation of the phenotype into variation explained by region-methylation profiles and residual variation. In particular, it assumes that the phenotypic similarity between individuals is totally captured by the region-methylation similarity, after the ascertainment adjustment via the PCs.

To compare the ascertainment correction approach in the multiple-case family, Fuady et al. computed the average distance of the effect of the change in TG on the change in methylation from a combination of 3 approaches. The average distance between the secondary phenotype and the naïve approach is smaller when the CpGs are not associated with the primary phenotype MetS (0.169 vs 0.418). This suggests that using MetS instead of coronary heart disease as the primary phenotype captures a part of selection mechanism. For CpG sites associated with the primary phenotype MetS, it is necessary to use the more complex secondary phenotype approach. For the other CpG sites, both approaches should provide the same results. Zhao et al. found that inclusion of the top 4 methylation-derived PCs was important in controlling for unknown confounding. Without this adjustment, the distribution of *p* values was very biased from what would be expected under the null. In this analysis, the logit transformation did not seem to provide any improvements. Moreover, as a consequence of the increase in sample size, the family-data analysis identified more significant genes than the analysis using unrelated individuals.

### Modeling the family structure

Four contributors used the linear mixed effect model to account for familial relatedness. Fuady et al. and Wang et al. used this model to assess the relationship between change in methylation and change in lipid levels in the naïve approach and MMLT, respectively. Specifically, they used *lmekin* function provided by *coxme* package in R [2] to conduct the analysis. In MMLT [15], the outcome was the lipid change while the change in median methylation was treated as an independent variable. In the naïve approach, methylation data was treated as dependent variable and the lipid changes as an independent variable. Sarnowski et al. used the linear mixed effect model provided by the QTDT software [12] to perform parent-of-origin effect association analyses with TG levels at baseline, follow-up, or on the difference between baseline and follow-up. They modeled the phenotype of the offspring as a function of covariates and genotype by taking parental original of each allele into consideration. In the contribution of Zhao et al., a linear mixed effect model was used in the family-based VC-score test [26].

In the parent-of-origin association analysis, Sarnowski et al. found a paternal effect of rs301621-G on the pretreatment and posttreatment of TG difference. They also found a maternal effect of this SNP on cg10206250 methylation levels. The observed paternal effect of this SNP is induced by treatment. However, this effect is not mediated by DNA methylation at cg10205250.

### Analyzing unrelated individuals

To analyze unrelated individuals, Zhao et al. used PCEV [25], which seeks to identify the linear combination of outcomes that maximizes the proportion of the variance being explained by the covariate. They contrasted the method using the VC-score test [26], which reduces significantly the model degrees of freedom compared to standard multivariate regression models. They restricted the analysis to genes on chromosome 11. The VC-score approach identified 1 gene, *SPTY2D1*, which was significantly associated with HDL-C changes. Using the PCEV approach, 1 gene, *NAV2,* was significantly associated with TG changes.

### Theme 2: Multimarker versus single-marker tests

Single-marker EWAS, which model the association between a phenotype and each CpG site individually, are widely used but may be underpowered owing to the small effect sizes often seen in epigenetic studies [33] and the high multiple testing burden of DNA methylation arrays [34]. Six papers in our group explored multimarker methods to test the association between a phenotype and set of CpG sites (Lent et al..., Huisman et al., Wang et al., Zhao et al., Xu et al., and Wu et al).

These methods can be categorized into gene score approaches, collapsing methods, and combination of EWAS summary statistics. The authors used real and simulated data to compare these multimarker association tests to the standard single-marker EWAS and evaluated consistency of results from different multimarker approaches.

### Gene score approaches

One way to reduce the multiple testing burden with methylation array data is to create gene scores and perform 1 test of association per gene rather than 1 test per CpG site. Wang et al. implemented 1 gene score method, MMLT [15], using the real data set, whereas Huisman et al. tested several gene score methods using the simulated data set. Wang et al. did not find any genes where the change in median methylation before and after treatment was associated with the change in either HDL-C or TG. Huisman et al. found that gene scores derived from single-marker EWAS *p* values—minimum gene *p* value, sum of log *p* values, and sum of squared log *p* values—had more power to detect simulated associations than single-marker EWAS. Gene scores derived from single-marker EWAS estimates of effect—maximum absolute value, median absolute value, sum, and sum of squared estimates of effect—had lower power than single-marker EWAS and higher Type I error.

### Collapsing methods

Like single-marker EWAS, gene score methods model the association between a phenotype and a single summary measure of gene methylation. An alternative way to perform gene-based tests is to model the association between a phenotype and all CpG sites in a gene. We refer to these approaches, jointly modeling the association between a phenotype and methylation at a set of CpG sites, as collapsing methods. Four papers in our group used collapsing method approaches to directly model the association between a phenotype and set of CpG sites in the real data.

Xu et al. performed a score test to evaluate the association between simulated posttreatment methylation and TG in candidate genes [6]. All other collapsing method groups employed a VC-based approach. Wang et al., Wu et al., and Zhao et al. all applied a SKAT [16] or adjusted SKAT (ASKAT) [26] test to the real data, and Wu et al. proposed an extension to a VC-based approach, aSPU [18].

Zhao et al. restricted their analysis to genes on chromosomes 11 and 19 and employed 2 multimarker approaches, SKAT and PCEV [25], described in Theme 1. There was some overlap in the top results of the SKAT and PCEV approaches: *OR8H3* was in the top 5 genes associated with change in HDL-C for both methods, and *P2RX3* was in the top 5 genes associated with change in TG for both methods. Wang et al. also performed a SKAT test of association across the whole epigenome and identified 2 genes, *GZF1* and *C18orf19*, where change in methylation before and after treatment was associated with change in HDL-C after correcting for multiple testing with a false discovery rate.

Wu et al. first performed an EWAS to quantify the association between methylation and pretreatment TG at each CpG site. They then performed 2 aSPU tests per gene, once using equal weights and once using inverse CpG sites variance weights, and compared to the EWAS for pretreatment TG. Additionally, 1 aSPU test per gene using the inverse variance weights was performed with change in methylation before and after treatment as the outcome. Using the inverse of the CpG site variance as the CpG site weights, Wu et al. identified 1 gene, *CPT1A*, associated with pretreatment TG after Bonferroni correction. This gene was also identified in the single-marker EWAS, but was not identified by aSPU when using equal CpG site weights.

### Combination of EWAS summary statistics

Lent et al. compared 1 novel method of combining EWAS summary statistics to perform regional tests, GlobalP, to 3 existing methods: Bumphunter [8], comb-p [9] and DMRcate [10]. For GlobalP, 178,015 regions were defined from gene and CpG island annotations. A test statistic for each region using the EWAS z-statistics was calculated, taking into account partial between CpG site M-values, and region *p* values were corrected for multiple testing using a false discovery rate. There was no overlap in regions identified by Bumphunter and other methods. GlobalP and comb-p identified regions in *CPT1A* associated with TG both before and after treatment. The single-marker EWAS also identified an association in *CPT1A*.

### Theme 3: Combining SNPs and DNA methylation probes

Three papers in our group combined both SNPs and DNA methylation probes (Huisman et al......., Sarnowski et al., Xu et al) to perform association analyses. One paper was an application of an existing method, the QTDT [12], in the real GAW20 data set (Sarnowski et al) and 2 papers proposed new methods (Huisman et al., Xu et al). In this section, we address 3 different questions: (a) Why use both SNPs and DNA methylation probes? (b) How to combine both SNPs and DNA methylation probes? and (c) What lessons did we learn from using both SNPs and DNA methylation probes?

### Why use both SNPs and DNA methylation probes?

Sarnowski et al. jointly analyzed SNPs and DNA methylation probes to better understand the biological mechanisms underlying parent-of-origin effects. Huisman et al. compared the performances of novel multimarker methods versus single-marker test using statistics based on SNPs or DNA methylation probes. Xu et al. developed 3 strategies to search for both genetics variants and CpG site variants associated with a quantitative trait (TG or HDL-C) in multiple genes: an iterative regression (single-SNP or single-CpG-based method), an extreme values approach (gene-based method), and a hybrid approach.

### How to combine both SNPs and DNA methylation probes?

We identified 2 common steps in the 3 papers: (a) a filtering of the number of SNPs and probes (CpG sites) to be tested and (b) a joint test of SNPs and DNA methylation probes.

#### Filtering of the number of SNPs and DNA methylation probes

One statistical reason for the filtering of the number of SNPs and DNA methylation probes is to reduce the number of SNP-CpG pairs tested and address the multiple testing issue. Another biological reason is to study the effect and interaction of SNPs and probes that are located in a same region (*cis*-effects). This first step was done based on either (a) GWAS and EWAS results (Sarnowski et al...) or (b) candidate genes (Huisman et al., Xu et al).

Sarnowski et al. tested the association of SNPs with TG under parent-of-origin effects using QTDT and selected suggestive associations based on an agnostic approach or a candidate approach (GWAS regions reported associated with TG). Then they selected CpG sites located nearby the suggestive SNPs (±50 kb) and tested the association with TG. Finally, they selected nominally associated CpG sites and performed a causal inference test [13] for each SNP-CpG pair. Huisman et al. selected candidate genes based on GAW20 simulation data. They defined 3 distinct groups of genes: 5 major effect genes with a causal SNP with high heritability, 34 minor effect genes with a causal SNP with modest heritability, and 39 randomly selected noncausal genes with no causal SNPs. For a genetic variant that was causal according to the GAW20 simulation [2], they selected the causal DNA methylation probe provided by the solutions, whereas for a noncausal genetic variant they selected the closest DNA methylation probe. Xu et al. used 2 different filtering strategies. In their iterative regression approach, few candidate SNPs and/or CpG sites highly correlated with trait values were tested first and a best variant was selected. The regression was repeated against the residual to select additional SNPs and/or CpG sites. In their extreme values strategy, the individuals with the top 5% value of the quantitative trait were used to select candidate genes with at least 1 CpG site.

#### Joint test of SNPs and DNA methylation probes

The second step was a joint test of SNPs and DNA methylation probes. Joint tests that combine both SNP and methylation data were used by Sarnowski et al. and Huisman et al.:

1 $$ CpG=\beta \mathrm{SNP}\ast SNP+\beta \mathrm{TG}\ast \Delta  TG $$

2 $$ \Delta  TG=\beta \mathrm{S}\mathrm{NP}\ast \mathrm{S} NP+\beta \mathrm{CpG}\ast CpG $$

Sarnowski et al. used eqs. (1) and (2) in the causal inference test to determine if a CpG probe mediates the association between a SNP and TG under a parent-of-origin effect model. Huisman et al. used test (2) as a data preprocessing step to get beta coefficients statistics or *p* values (for SNP and CpG sites) to aggregate in their multimarker methods. For each gene, they created 4 scores by aggregating beta coefficients or *p* values for SNPs or DNA methylation probes (sum of absolute values, sum of squares, maximum of absolute values, and median of absolute values in the spirit of other papers exploring aggregation methods [35–37]). Xu et al. used a multimarker approach to test the combined SNPs/CpG sites or candidate genes from the iterative regression and the extreme values strategy (a score test developed by Chapman et al. [6]). They compared the performances of the different approaches (iterative regression, extreme values strategy, or a hybrid approach) with the correlated method [23].

### What lessons have we learned from the GAW20 data set when using both SNPs and DNA methylation probes?

Sarnowski et al. identified 22 SNPs with suggestive parent-of-origin effects on TG (*P* ≤ 10^− 5^) and 18 DNA methylation probes located nearby these SNPs were found associated with TG (*P* ≤ 0.05). One SNP-probe pair presented evidence of parent-of-origin effect: the SNP rs301621 was associated with the difference between pretreatment and posttreatment TG when transmitted by the father (*P* = 1.2 × 10^− 5^). This same SNP was associated with the methylation levels of cg10206250 when transmitted by the mother (*P* = 0.01). Using a causal inference test, the authors showed that the observed parent-of-origin effect of rs301621 was not mediated by DNA methylation at cg10206250. Huisman et al. performed gene-based tests based on SNP or CpG estimates from the joint test of SNP and CpG on TG. They found an average power of 0.48 and a Type I error of 0.04 when using the SNP statistics and a power of 0.06 and a Type I error of 0.04 when using the CpG statistics. Their results also suggested that methods run on major effect genes (with causal SNPs) were detecting more “signal” than on minor effect genes (with no causal SNPs). Finally, they found that single-marker tests outperformed gene-based tests in general. Xu et al. found that the correlated method and the hybrid approach had correct Type I errors for TG association analyses, but were conservative for HDL-C. The other methods were more conservative for both traits. The patterns of power comparison for TG and HDL-C were consistent. From the most powerful to the least powerful, the methods were the hybrid approach, the iterative regression strategy, the extreme values strategy, and the correlated method.

## Discussion

Numerous methodological questions exist about how best to analyze genome-wide methylation data. However, based on 8 individual contributions investigating related questions about analysis strategies, we have identified a few preliminary recommendations and conclusions.

First, as it is the case for GWAS, adjustment for familial relationships appears to be both standard practice and an effective approach for methylation studies. Accounting for ascertainment bias, however, appears less mainstream, though it has a potentially measurable effect on conclusions. Further exploration of methods and impact on analyses is suggested.

Second, the use of multimarker methods appears promising as a way to reduce multiple testing penalties, aggregate effects, and provide more precise biological interpretation. Further work is needed, however, to identify an optimal or “gold-standard” set of multimarker methods to become part of a standard analysis pipeline for EWAS. Currently, numerous methods are available and a comprehensive understanding of the pros and cons of these methods remains elusive.

Third, the simultaneous analysis of methylation data and genetic markers also appears promising, as a way to both uncover true biological mechanisms and improve statistical power. However, it is worth noting that the integration of multiple data sources brings with it the challenge of knowing how to best integrate multiple types of data. Precise specification of statistical models and how they reflect biological mechanism is important. It remains easy to grab an off-the-shelf method that results in unexpected behavior. We remain optimistic, however, that such approaches are both necessary and hold great promise for the future of genetic epidemiology.

Fourth, the field of genetic epidemiology continues to wrestle with the promise of ever larger data sets that are pitched to provide enough data so that a lack of statistical power should no longer be an excuse for an inability to identify causal relationships. However, as is evidenced by the GAW20 workshop data sets, data sets with “only” hundreds to thousands of individuals who have a complete set of phenotypic and genetic measurements remains an acute reality. With this mind, methodological innovations, which seek to maximize biological interpretation and statistical power despite complete data on only hundreds or thousands of individuals remains an important area of future research.

## Conclusions

### Conclusions of theme 1

Three conclusions can be drawn from theme 1. First, modeling family data using a kinship matrix is now standard practice. Second, an ascertainment correction for multiple-case families and unrelated individuals seems to be necessary, although there may be no agreement on how best to do them. Finally, pedigree information may be useful for identifying parent-of-origin effects.

### Conclusions of theme 2

In the simulated data, Xu et al. and Huisman et al. found that multimarker methods had modestly increased power to detect epigenetic associations compared to single-marker methods. The simulated epigenetic effects in the GAW20 simulated data set were not multimarker effects, so the increase in power was likely owing to a reduced multiple-testing burden, indicating that multimarker methods are useful even without multimarker effects. Huisman et al. also found that multimarker tests based on effect size had increased Type I error.

In the real data, Wang et al. and Zhao et al. found that multimarker tests identified associations that single-marker EWAS did not. There was little overlap in results between groups because of the different choices in time points and phenotypes modeled. However, Lent et al. and Wu et al. both found that *CPT1A* was associated with TG before treatment. DNA methylation of this gene was previously implicated in lipids [38, 39].

The increased power in the simulated data set, difference in findings between single-marker methods and multimarker methods, and replicated multimarker results in the literature give evidence that multimarker methods are useful to increase power to detect epigenetic associations.

### Conclusions of theme 3

The causal inference test is an appealing approach to discover new mechanisms of action of SNPs, particularly in previously TG-reported loci. It can be a promising method for further understanding candidate EWAS associations. There is a possibility for wider use and application of gene-based tests to summarize SNP and CpG estimates when analyzing methylation data.

## Abbreviations

aSPU: Adaptive sum of powered score
CpG: Cytosine-phosphate-guanine
EWAS: Epigenome-wide association studies
GAW20: Genetic Analysis Workshop 20
GMMAT: Generalized linear mixed model association test
GOLDN: Genetics of Lipid Lowering Drugs and Diet Network
GWAS: Genome-wide association study
HDL-C: High-density lipoprotein cholesterol
MetsS: Metabolic syndrome
MMLT: Median methylation level test
PC: principal component
PCEV: principal component of explained variance
QTDT: Quantitative transmission disequilibrium test
SKAT: Sequence kernel association test
SNP: Single nucleotide polymorphism
SOLAR: Sequential Oligogenic Linkage Analysis Routines
SPU: Sum of powered score
TG: Triglyceride
